# Keeping Teachers Engaged during Non-Instructional Times: An Analysis of the Effects of a Naturalistic Intervention

**DOI:** 10.3390/educsci13060534

**Published:** 2023-05-23

**Authors:** Katherine Bateman, Sarah Emily Wilson, Katherine Matthews, Ariane Gauvreau, Maggie Gucwa, William J. Therrien, Rose Nevill, Micah Mazurek

**Affiliations:** 1 College of Education, The Haring Center for Inclusive Education, University of Washington, Seattle, WA 98195, USA; 2 Wested, San Francisco, CA 94107, USA; 3 School of Education and Human Development, University of Virginia, Charlottesville, VA 22904, USA; 4 The Faison Center, Richmond, VA 23230, USA

**Keywords:** teaching training, naturalistic intervention, social skills, universal design for learning

## Abstract

As the prevalence of autism continues to rise, early childhood programs continue to evolve to meet the needs of children across a spectrum of abilities. To do this, strategies and supports are needed for teachers to engage with children who experience difficulties across developmental domains. Snack Talk, a naturalistic visual communication intervention, focuses on increases in conversation engagement for children with autism and related disabilities during mealtimes. This study examined the effects of the implementation of Snack Talk on increasing teacher engagement in conversation with five preschool children with autism during mealtimes in an EIBI classroom setting. A reversal design was used to analyze the relationship between Snack Talk and teacher conversational engagement with children. Results of this study demonstrated that implementation of Snack Talk increased instances of teacher engagement in conversation with children compared to baseline phases, demonstrating the promise of this intervention supporting students’ different levels of support needs in inclusive, blended settings. A functional relationship has been established between baseline and intervention phases and generalization. Limitations and directions for further research are discussed.

## 1. Introduction

The prevalence of autism spectrum disorder (ASD) continues to rise in children [1], changing the landscape of early childhood classrooms that many children are served in as education systems push for inclusive, blended settings designed to provide students of all abilities with an effective, meaningful education. The vast array of abilities present in individual children with ASD requires implementation of many evidence-based practices to ensure every child meets their specific goals and accesses a free and appropriate education. Ensuring that educators supporting these children have access to high-quality professional development and training is essential to ensure rigorous implementation of these practices.

### 1.1. Inclusive, Blended Classroom Settings

A robust literature base identifies positive outcomes of inclusive, blended classrooms for children with and without disabilities [2–6]. Among others, these outcomes include increased academic achievement, strong relationships within the classroom, as well as community membership and growth of overall classroom community [3,5,7–9]. Yet, simply placing children with and without disabilities in the same classroom does not automatically ensure inclusion and the associated outcomes. Rather, inclusion requires thoughtful, smart planning and instruction to ensure that all children are able to access their education [8,10]. This differentiation is important, as it is the difference between integration of students with disabilities into a general education classroom and inclusion of students with disabilities into a general education classroom.

Interventions designed universally to meet the needs of a spectrum of ability levels are crucial to the success of inclusive, blended settings. To promote learning for all students in one setting, implementation of numerous strategies and supports are necessary, specifically naturalistic interventions that can easily be embedded within instruction to ensure all students are engaged and learning [11].

### 1.2. Naturalistic Interventions

Children with ASD often require interventions to address differences in social communication and language development. Naturalistic interventions, which are embedded in the natural milieu and routines of the classroom, are considered an evidence-based practice to develop the social communication and linguistic skills of children with ASD [12]. Naturalistic interventions are a set of techniques and strategies that include interventions such as milieu teaching [13], incidental teaching [14], and pivotal response training [15,16] along with prompting, visual supports, and reinforcement.

Naturalistic interventions were developed to increase children with disabilities’ access to, and engagement in, inclusive environments while providing individualized support [9,17–20]. These strategies have been particularly promoted within early childhood special education (ECSE) and early intensive behavioral intervention (EIBI) settings and are embedded within the child’s regularly occurring activities and routines. Naturalistic interventions focus on supporting increasingly complex skills needed by children to engage in those activities and routines while leveraging the child’s interests and the arrangement of the child’s environment [21].

### 1.3. Social Skills Instruction

As a whole, naturalistic interventions are designed to promote motivation, functional relationships, and skill generalization [22]. This is particularly meaningful when considering the usage of naturalistic interventions to facilitate skills such as communication and social skills instruction as social skills interventions have historically been associated with a lack of generalization and limited effectiveness [23], attributed to the tendency for social skills training to occur in “contrived, restricted, and decontextualized” [24] (p. 340) environments.

When targeting social communication and language outcomes, naturalistic interventions often involve environmental arrangements designed to foster engagement of learners as language partners [7,25] while adults evoke, model, and reinforce novel language usage within functional contexts [26] to support a social feedback loop. Support of a social feedback loop has been theorized to be especially critical for children with ASD who already experience language and communication difficulties [27,28]. In several studies, young children with ASD were found to emit fewer overall vocalizations and fewer speech-related vocalizations when compared to their typically developing peers [27,28]. This reduction in vocalizations was associated with decreased opportunities for engagement in conversation and social interactions with peers as well as decreased responsiveness and engagement from adults [27,28]. This is concerning as a lack of adult engagement in reinforcing children’s vocalizations within the feedback loop may further decrease children with ASD’s access to and engagement in conversations necessary for language development. As such, research exploring effective naturalistic interventions for supporting both child and adult responses within a social feedback loop is critical.

### 1.4. Teacher Training

A gap persists between effective naturalistic and social skills interventions for young children and the training teachers receive to implement these strategies. Unfortunately, studies exploring both pre-service and in-service teacher training and social skills demonstrate a lack of preparation in both areas [29–31]. One of the most essential components of teacher training is real-time teaching experiences. These experiences, often called field experiences, include authentic contexts where pre-service teachers implement the theory of teaching [32] and are some of the most influential aspects of a teacher’s preparation [33]. Through field experiences (also called “practicum”, “student teaching”, “internships”, and “fieldwork”), pre-service teachers can observe a skilled mentor using effective practices, develop course-to-field connections, engage and collaborate with other educators, and implement instructional strategies they have learned in coursework [32,34,35]. However, extant literature suggests that pre-service and in-service special education teachers do not consistently receive sufficient training to support the social development of children through social skills instruction [29,30].

Teacher education curriculum outside fieldwork experiences emphasizes behavior management techniques and supporting the behavior of students [36] rather than intensive instruction on the instruction of social skills [30]. A national survey showed that while teachers believe social–emotional learning and social skills intervention is important, they report limited training and confidence in supporting students with these skills [29]. Dobbins and colleagues [30] surveyed licensed teachers (*n* = 237; 150 special education teachers and 87 general education teachers) across nine colleges of education. Participants were asked to complete a questionnaire regarding the level, type, and area of social skills instruction they experienced during their pre-service and in-service teacher education programs. Of the 150 special education teachers, 28% reported receiving no social skills instruction during their teacher preparation program while 46% reported no social skills instruction during their in-service training program. Therefore, it is likely that pre-service teachers are learning from experienced mentor teachers who have had little social skills training themselves. This cycle of inadequate training and lack of implementation of meaningful social skills interventions continues as new teachers enter the workforce with insufficient training and minimal opportunities to learn effective teaching strategies and supports in this area of instruction. As a result, educators across the country miss the mark in providing this important instruction to their students.

A contributing factor to inadequate training in social skills instruction for teachers is when educators do implement social skills instruction, research indicates that they are more likely to use class-wide interventions [37]. As such, social skills interventions that are easy to implement with fidelity and that benefit a range of students with different needs should be prioritized. Further, attention should be given to the development of interventions that allow for easy staff training and support staff engagement in naturalistic interventions with children during established routines.

### 1.5. Snack Talk

One typically occurring classroom routine with potential for naturalistic and embedded social skills instruction is mealtime. Mealtimes are a consistent routine across educational, home, and community contexts [38]. In school settings, children may be motivated to develop social communication skills [39] within this routine as these skills are needed by children to participate fully with their proximally seated peers. Alone, this natural routine may encourage neurotypical children to engage in reciprocal conversations [39], but children with ASD and related disabilities often need additional teacher-led supports and instruction to increase their access and meaningful participation in these interactions [40].

One example of a naturalistic intervention that is embedded in mealtime is Snack Talk [7,25,41,42]. In this intervention, a visual support intervention package (i.e., modeling, prompting, and reinforcement) is used during the natural routine of mealtime to prompt social exchanges and conversation among children. Snack Talk visuals are laminated pages with 9–12 photos or symbols that are related to a question prompt at the top of the page. The visual supports are created around age-appropriate conversational topics that are of interest to participating children. For example, the Snack Talk card could say, “What do you like to do at recess?” and depict the favorite recess activities of several students within the classroom, motivating children to engage socially with each other using the same Snack Talk card. The visual is implemented by the teacher who models asking the question prompt then answering by pointing to a picture on the card. As the teacher shows the cards to the students during mealtime and gives the students opportunities to respond and interact with each other, the mealtime becomes interactive at a level that is individualized and meaningful to the students. In doing so, teachers increase students’ opportunities to respond while also increasing their own engagement with students. Although the Snack Talk procedure is aimed at increasing the engagement of students, the interactions it occasions among the teachers are an equally important outcome. As such, we examined the efficacy of Snack Talk in supporting and increasing the conversation engagement of five preschoolers with each other [7] as well as the effects of the implementation training and intervention on the teacher engagement with these children. Data regarding child-level outcomes of the implementation of Snack Talk procedures in an early intervention preschool classroom have been presented and discussed in Bateman and colleagues [7]. This paper presents teacher-level outcomes of the implementation of Snack Talk procedures in an early intervention preschool classroom. To this end, the following research questions were addressed in this study:

1. What are the effects of the implementation of Snack Talk on teacher engagement with preschoolers with disabilities during mealtimes?
2. What are teachers’ perceptions of the implementation of Snack Talk?

## 2. Method

### 2.1. Participants and Setting

This study occurred in an urban, early intervention preschool classroom in the Mid-Atlantic that provided Applied Behavior Analysis [43] to children diagnosed with ASD and other developmental disabilities [7]. This setting was chosen for participating in this study because of its high-quality EIBI program. At the time of the study, 11 children attended the preschool, supported by 15 teachers and staff members. All teachers and staff members were responsible for providing 1:1 individualized and small group instruction, as well as supporting students during non-instructional times. A comprehensive application of behavior analysis was used in this classroom, in which strategies derived from research in ABA were applied both to student instruction and teacher training [44]. All teachers were required to complete a minimum 2-week introductory training in ABA as well as demonstrate competency in applying practices rooted in ABA through regular observations by their supervisor, prior to working with students. On a typical day, the teachers would be assigned 1 h blocks of instruction with students such that through the course of a 6 h instructional period, they might provide instruction to 6 different students. The frequent rotations were part of the classroom design to ensure a strategy to naturally program and test generalization.

Five children and fifteen adults participated during the course of the study. The children remained constant throughout the study, but staff were rotated among children throughout the course of the study, increasing training for generalization from the onset of intervention [45,46]. Teacher participants included in this study had to be employed in the preschool classroom and be a Registered Behavior Technician (RBT) or have a higher level of credentials (i.e., Board Certified Assistant Behavior Analyst [BCaBA], Board Certified Behavior Analyst [BCBA], or Board Certified Behavior Analyst-Doctoral [BCBA-D]). Teachers must have received school-wide training in ABA and early intervention and attended the Snack Talk intervention training. Children that participated in this study met the following criteria: (a) have a medical diagnosis of ASD, which was an enrollment requirement for the early education center, (b) a chronological age between 3 and 4, (c) be eligible for early intervention services at the early education center, and (d) have identified challenges in social communication as indicated by a teacher report, as reported in Bateman and colleagues [7]. Teachers and children participating in this study did not receive any incentives for participation.

#### 2.1.1. Teacher Participants

During the course of this study, 15 teacher participants acted as 1:1 implementers of intervention. Teacher participants were intentionally rotated daily in their assignment of supporting the child participants. Teacher participants included 2 team leaders who were Board Certified Behavior Analysts (BCBAs), 12 teaching assistants, and 1 part-time teaching assistant. The 12 teaching assistants included 2 graduate students who were in ABA programs and 10 RBTs. One of the team leaders was Black and the other team leader was White. Both were females who held Bachelor’s degrees. At the time of the study, both had over 4 years of experience working with children with ASD and other developmental disabilities. Of the teaching assistants, 2 were Black or African American, 1 was Native Hawaiian or Other Pacific Islander, and 9 were White. Of the 13 teaching assistants, 12 were female and 1 was male. Teaching assistants held a range of degrees including 4 teaching assistants with high school diplomas, 3 teaching assistants who were working towards the completion of their Bachelor’s degrees, and 5 teaching assistants who held a complete Bachelor’s degree. They had a range of years of experience working with children with ASD and other developmental disabilities that spanned between 6 months and 3.5 years. The part-time teaching assistant was an African American female, with a high school diploma, and 4.5 years of experience working with children with ASD and other developmental disabilities.

#### 2.1.2. Preschoolers Accessing Snack Talk

Corey, Marie, Jonathan, Claire, and Justin (all pseudonyms) were between the ages of 3 and 4 during the time of the study [7]. All students had a medical diagnosis of ASD with Corey having an additional comorbid diagnosis of Receptive/Expressive Language Delay. Students received 6.5 h of weekly ABA services. Marie, Claire, and Justin received additional speech therapy support and Jonathan received additional occupational therapy support. Corey, Marie, Jonathan, and Claire are all monolingual English speakers. Justin is bilingual and his family speaks Spanish predominantly in their home. At the time of the study, Corey and Justin were considered pre-emerging in their speaking and listening skills with Marie, Jonathan, and Claire considered to be emerging speakers and listeners. Parental consent was obtained for all children participating in this study. Additional information on child participants can be found in Bateman and colleagues [7].

### 2.2. Materials

During intervention implementation phases, staff implemented Snack Talk [7,25,41,42], a visual-based naturalistic intervention designed to promote social communication within the natural conversational routine of mealtimes. Snack Talk cards were created by the research team with input from implementing staff at the preschool. Snack Talk cards were developed around the children’s highly preferred topics and interests and were printed on 8 ½-by-11-inch laminated sheets of paper. They included a socially based topic question (i.e., “What movie do you like”?) and multiple visuals as possible answers to the target question [41,42]. Implementation and least to most prompting procedures based on the staff training were affixed to the back of the cards. Cards were created using Microsoft Word^®^ and a Google^®^ Image search under a Creative Commons license. A video camera and tripod were used to collect data during all phases of the intervention, and a laptop was utilized for data coding and analysis.

### 2.3. Behavioral Definitions and Measurement

Data collection occurred during the first 10 min of each mealtime which were video recorded and coded using a 10 s partial interval recording procedure to identify teacher engagement with children during mealtimes. Data were collected on all teachers working with child participants. Coders were two undergraduate research assistants who were trained by the first author. Coders used a dependent variable data collection form to record if an instance of teacher engagement with a target child occurred at any point during the 10 s interval for the duration of the mealtime video. As this naturalistic intervention was designed and implemented as a class-wide intervention with rotating teacher assignments, teacher engagement was measured and reported in aggregate with classroom-level data serving as the unit of analysis. Teacher engagement was measured by calculating the averaged percent of overall staff engagement per child across staff members. This was done to assess the effect of the training and intervention materials on overall teacher engagement with children while accounting for changes in the number of teachers due to child absences.

Teacher engagement was defined as a verbal or nonverbal conversation initiation or response by the teacher to a child during mealtime. Initiations and responses were coded as engagement regardless of topic or use of the Snack Talk card. For example, a teacher asking a child to open their lunch, responding to a Snack Talk card, or responding to a request from a child were all considered teacher engagement. Teachers talking to children not in the study or another teacher were not considered teacher engagement and were therefore not coded.

Although partial interval coding procedures tend to overestimate engagement in the target behavior(s) [47], this procedure was selected to account for the natural lulls and breaks in conversation engagement during mealtimes.

### 2.4. Experimental Design

This intervention utilized an ABAB withdrawal design [48,49] for all participants. This methodology was designed to meet the criteria for single-case research design standards [50] in accordance with What Works Clearinghouse. Due to the nature of this naturalistic group intervention, teacher and child participants moved through baseline and intervention phases together as intervention was provided by support staff to participating children simultaneously during the midday mealtime. The staff supporting each child changed throughout the intervention to support skill generalization [45,46]. Phase changes occurred after at least 5 days of data collection in each phase in alignment with guidelines for single-case research design [50]. Some phase changes occurred after more than 5 days to account for school holidays and events.

### 2.5. Procedures

During all study conditions, the established lunchtime routine was maintained. Children who were not consented to participate in the study were seated separately during all phases. Sessions across conditions began once all children and teachers were seated at the table with 5 teachers supporting 5 children. Teacher participants supported focal children in a 1:1 or 1:2 ratio. All children worked with all teacher participants during the study. In both conditions, coding procedures continued if a child left the table. Data collection intervals continued to be coded in these instances as nonengagement on the part of the student and therefore nonengagement on the part of the supporting teacher. Prior to beginning the initial baseline condition, verbal and attention-based social reinforcement was identified by teachers as an effective form of reinforcement for all participants and was used throughout the duration of the study.

#### 2.5.1. Baseline Sessions

During baseline sessions, the mealtime routine was conducted as business as usual as mealtime was a pre-established, familiar routine in this classroom. This includes the mealtime routine and the level of interaction between teachers and children. Children participants sat at the table to eat lunch with staff providing support from behind. Snack Talk supports were not provided in this condition to children or staff and teachers were directed to interact with the child participants as they would typically do.

#### 2.5.2. Teacher Training Procedures

Prior to the start of the intervention, teachers received training in intervention procedures by the first author. This teacher training followed evidence-based procedures [51]. This protocol included the following steps: (1) describe the target skill, (2) provide a succinct written description of the target skill, (3) demonstrate the target skill, (4) practice the target skill, (5) provide feedback during practice, and (6) repeats steps 4 and 5 until skills mastery [51]. Before the onset of intervention, all teachers demonstrated 100% fidelity on the steps of implementation. During the implementation of the intervention, teachers received on-going, weekly training to maintain high fidelity of implementation.

#### 2.5.3. Intervention Sessions

During intervention sessions, staff implemented the full Snack Talk protocol as described by Bateman and colleagues [7]. In the intervention conditions, multiple Snack Talk cards were placed on the table by the teacher participants where they were easily available to the child participants. Teacher participants then modeled engaging in conversation using the Snack Talk card. First, the teacher engaged in a group model by identifying what they liked on the card such as “I like to go to the park on the weekend! Where do you like to go?” The teacher then paused for 5–10 s to allow child participants an opportunity to independently and naturally respond. If the child participant engaged naturally, the teacher provided verbal social praise as reinforcement and asked each child the topic question on the Snack Talk card. After the group model, teachers began prompting children to use the Snack Talk cards with their peers, providing individualized levels of prompting as needed. The group model was provided each session for the duration of the intervention conditions.

In the case that child participants did not respond to the group model to initiate conversation, or respond to a peer’s question, teachers used least-to-most prompting to facilitate conversation engagement. Least-to-most prompting was also used during lulls in the conversation to encourage children to engage with the Snack Talk cards and with each other. For the purpose of this study, a natural lull in conversation was defined as a period of more than 10–15 s where children did not engage in verbal or non-verbal communication towards a teacher or peer. Teachers first used gestural prompts (i.e., pointing) followed by verbal prompts (e.g., “He asked what you like to eat” or “Let’s ask a friend what they like to do on the playground”) as needed for non-response. As needed, teachers moved up the prompt hierarchy until children engaged independently with the Snack Talk cards, their teachers, or peers. Regardless of prompting level, reinforcement in the form of social praise was immediately provided when a child engaged in conversation. These procedures were used for the entirety of the mealtime.

#### 2.5.4. Follow Up

After the final intervention condition, implementation of Snack Talk protocols continued with the entire class. Follow up generalization data were collected on teachers utilizing Snack Talk with Corey, Marie, Jonathan, Claire, and Justin. A total of three follow-up data points were collected every two weeks across a duration of six weeks. In these probe sessions, teachers implemented Snack Talk supports identical to intervention phases and data were collected using 10 s partial interval recording procedures.

### 2.6. Procedural Fidelity

Procedural fidelity was assessed via video observations. Assessors watched video recordings of each session during all study conditions to code consistent implementation of intervention using a procedural fidelity checklist developed by the first author. The checklist consists of 6 steps of intervention: (1) place Snack Talk on the table during mealtimes; (2) model by making an on-topic comment and showing the table the Snack Talk card; (3) if students respond, reinforce these interactions with social praise and other individualized reinforcement systems if appropriate; (4) if students do not respond, begin least to most prompting and continue the prompting hierarchy until the target response occurs; (5) reinforce as target responses/initiations occur; and (6) as a natural lull in conversation occurs, begin prompting hierarchy again. Data were collected and coded across all phases of the intervention. Procedural fidelity was measured for all teacher implementers and results indicated procedures were followed to 100% fidelity across all implementers of intervention during intervention conditions. During the baseline conditions, procedural fidelity was 0% as the Snack Talk intervention protocol was not implemented.

#### Interobserver Agreement (IOA)

A team of trained undergraduate research assistants who were monitored by the first author completed coding for the dependent variable. Two undergraduate research assistants served as independent IOA coders. Before beginning IOA coding, the two undergraduate research assistants were trained to 90% mastery criteria using interval-by-interval IOA procedures [52]. Once consistent agreement was reached, randomly selected 25% of sessions in each condition for each child participant were coded for IOA. Design standards for IOA were met [50] as IOA averaged 94% across all participants and conditions, ranging from 83% to 100%. Table 1 reports the condition and child-specific IOA.

### 2.7. Social Validity

Perceptions of the intervention’s social validity were measured through anonymous, self-report surveys collected from the teacher participants [7]. Teacher participants were asked 4 questions and were provided space for any additional feedback regarding effectiveness of the intervention and ease of implementation. Surveys were distributed by the research team to the classroom supervisor who distributed the surveys to the teacher participants. Once completed, the classroom supervisor collected and securely stored the anonymous surveys. Surveys were then collected from the classroom supervisor by a member of the research team who analyzed the results.

## 3. Results

### 3.1. Teacher Engagement

For all participants, the aggregate level of teacher engagement with children during baseline data collection phases was dramatically low. Teachers engaged with children at low levels, ranging from 3% to 15% of intervals. During intervention phases, immediate increases were demonstrated in the level of teacher engagement, ranging from 15% to 24% of intervals. While data demonstrated overall stability across all phases, ranging from 12% to 9% in variability, the first data point in both baseline phases stands out. These data points are higher than the rest of the data collected in each of these phases. This may be attributed to the reactivity of teachers during video data collection. Following day 1 of each baseline phase, data demonstrated a decrease in teacher engagement, suggesting habituation to video data collection.

**Figure 1.**
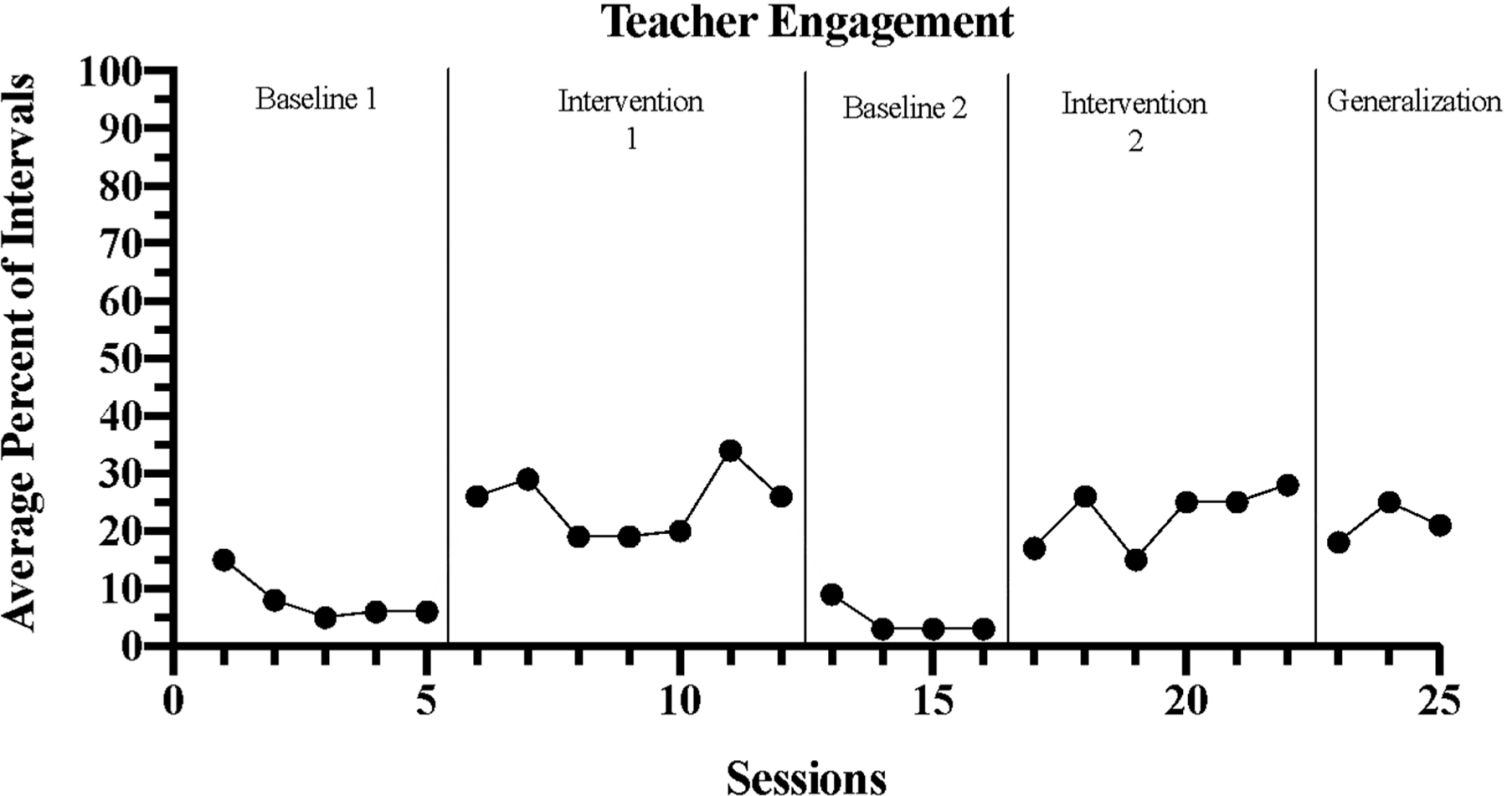
Percent of intervals teachers engaged in conversation.

Minimal overlap occurred between intervention phases. In comparison to baseline phases, intervention and generalization data showed one overlapping data point, indicating that the percentage of nonoverlapping data (PND) was 96%. Data collected in generalization probes suggested a maintained effect, demonstrating a consistent, increased level of teacher engagement with children during mealtimes. Overall, these data indicate that when Snack Talk was implemented, teachers engaged at higher rates with children during mealtimes. The results of the average level of teacher engagement are shown in Figure 1.

### 3.2. Social Validity

Social validity results indicated that all teachers found this intervention to be effective at increasing their engagement with children during non-instructional times, such as mealtimes. When asked for any additional feedback on the Snack Talk intervention, teachers identified that the implementation of Snack Talk supports during mealtimes helped staff engage with the children at the meal table and provided them with clear procedures to follow during a time in which they identified they previously engaged minimally. Teachers rated the intervention as highly effective in increasing students’ overall engagement during mealtimes as well as the focal students’ repertoire of conversational topics. See Table 2 for social validity results.

## 4. Discussion

The primary purpose of this study was to determine if the implementation of Snack Talk intervention procedures increased engagement and interactions among children and teachers during a non-instructional time. This study builds on extensive evidence from previous studies [7,25] that demonstrates that the Snack Talk intervention protocol promotes social interactions, specifically among teachers and children in this application. Further, the current study extends findings in previous Snack Talk studies [7,25], demonstrating that the intervention also increased teacher engagement with children, emphasizing the effectiveness and applicability of this intervention to teacher and staff engagement during non-academic times. Results demonstrated differences in teacher engagement when the Snack Talk procedure was in place and when it was not. Creating opportunities to target social interactions during mealtimes ensured additional moments where teaching and learning could occur in an early intervention program. Given the importance of social interactions for students with ASD and the occurrence of natural social opportunities presented during activities such as mealtimes, this finding is meaningful for children as it highlights the dual function of improving teacher behavior while increasing social interactions among children.

This naturalistic, simple intervention provided teachers and staff with a protocol to engage with children during a commonly known down-time in classrooms, increasing children and teachers’ opportunities for social engagement during mealtimes. This study affirms prior research examining the benefit of naturalistic interventions in increasing access to and engagement in inclusive settings [9,17–20,22]. Further, with explicit initial training and clear, simple steps of implementation, teachers were able to achieve high fidelity of intervention implementation. Because of its ease of implementation, Snack Talk has the potential to help increase teachers’ efficacy in supporting social skills development in children. This is important as findings from extant literature suggest that pre-service and in-service special education teachers do not receive sufficient instruction in supporting the social skills development of children [29,30]. Snack Talk served as a prompting procedure for teachers to engage with their students, maximizing opportunities for instruction and capitalizing on social conversation.

Having a procedure that is easy for teachers to implement is important for two reasons: (1) teachers are not accessing formal, dedicated social skills training in teacher preparation programs and therefore are less likely to know how to develop or implement social skills instruction, and (2) the simplicity of the procedure supports quick engagement among the teachers and students which naturally increases the availability of reinforcement for teachers during implementation. When teachers feel successful, implementation of intervention may occur at higher rates, resulting in overall positive outcomes for teachers and students during mealtimes. Following the disruptions in education training programs due to the COVID-19 pandemic, this is even more important. Teachers are entering the workforce with less in-person, hands-on experience as a result of remote learning in teacher preparation programs. This intervention is easy enough to implement that it creates opportunities for teachers to feel competent and confident in delivering interventions to children of all abilities, ultimately increasing teachers’ feelings of self-efficacy. This outcome is important, as EI and ECSE programs seek to increase learning and skill acquisition to ensure children are learning during all moments of their day, enhancing overall program effectiveness.

Finally, another promising aspect of these data relates to the way children develop language by supporting a social feedback loop [27,28]. Before conversing and interacting with peers, children first engage in conversation with the adults in their environment [27,28]. It is likely that this increase in opportunities to engage in communication with a familiar adult is a prerequisite skill to engaging in interactions with a peer. Further, anecdotal observations of Snack Talk studies in multiple contexts reflect that teachers are more likely to engage in mealtime conversation with children who already demonstrate more sophisticated communication repertoires [7]. Children with significant communication delays, dual language learners, and those using AAC devices are often left out of conversations as teachers with minimal training and experience may struggle to know how to engage using different modalities of communication. This study showed that Snack Talk provided teachers with a universal way of engaging with students who use a wide range of communication modalities, increasing the frequency of meaningful social interaction opportunities and making this intervention ideal for settings that include students of all abilities.

Serving children with a range of abilities effectively in classroom settings requires thoughtful planning, implementation, and continuous assessment and analysis to ensure learners can meaningfully access the curriculum and fully engage in their educational experiences. Providing inclusive education to students with and without disabilities is complex, incorporating not just the mission or vision of inclusion, but also the instructional practices needed to enact this vision [10]. Inclusion is built on the belief that all learners are active members of their community and have a sense of belonging within their classroom regardless of diagnosis or ability level [4,6]. To implement inclusion well, interventions designed with opportunities for differentiated instruction and engagement are needed to ensure all learners have equitable opportunities to access their education [10]. Snack Talk is an inclusive, universally designed intervention that promotes social communication, fostering social skills and an individual’s sense of social belonging through opportunities to engage socially with teachers and peers in their classroom. This study demonstrated the effectiveness of Snack Talk with children with diverse language and communication profiles, demonstrating positive outcomes of intervention with children with a range of abilities and communication modalities. These results are promising and indicate this intervention may be a good match in blended, inclusive classroom settings.

### 4.1. Implications for Practice

This study suggests several implications for practice in settings with learners of all abilities and language profiles. First, the low cost and high ease of implementation of this naturalistic intervention, combined with the incorporation of the universal design for a learning approach that is inherent in Snack Talk, produces outcomes that enhance the overall effectiveness of EIBI and ECSE programs. This intervention is easily embedded within an existing routine that supports skill development in an often-underutilized time for instruction. This allows teachers to focus on fostering important social communication skills during a routine that is inherently social. Second, using Snack Talk in classroom settings ensures teachers know what and how to engage with learners of all abilities and maximizes teaching and learning opportunities for children. Snack Talk cards provide teachers with a structured way to interact with students and reinforce a social feedback loop.

Next, this intervention can easily be altered based on the age and communication level of students. Teachers can implement this routine with children with a wide range of communicative abilities and individualize the prompts and pictures on the Snack Talk cards to meet the needs of all learners. While this instance of implementation of Snack Talk occurred in young children in an early intervention program, this intervention has been successfully adapted for use in secondary settings as well as day treatment centers for adults with disabilities [25]. To do so, teachers should survey students and caregivers to learn more about their interests and develop Snack Talk cards that reflect the individual students. This ensures students have preferred interests to talk about to their peers, increasing motivation and reinforcement to engage in intervention. This ability to easily modify interventions to different ages of individuals with disabilities is important as young children grow older into adults and attend social outings centered around meals.

Third, generalization data collected in this study demonstrated that the increased effects of the intervention were generalized following the intervention when Snack Talk was implemented. This highlights the ease of implementation without extensive training, materials, and preparation. These findings are important, as EI and ECSE programs grapple with issues related to teacher and personnel shortages exacerbated by the pandemic [53].

### 4.2. Limitations & Future Research

There are some limitations to this study that are important to consider. First, the educational setting primarily served children with disabilities and communication delays, which inherently resulted in the use of different modalities of communication (e.g., PECS, AAC), as children had a range of communication strengths and challenges. It is likely that child-to-child and teacher–child communication would look very different in a different classroom where some children do not demonstrate communication delays. Future research should explore the implementation of Snack Talk in different settings including children with more advanced communication abilities.

Another potential limitation is the teacher-to-student ratio. The setting was staffed for nearly a 1:1 ratio, which resulted in a highly scheduled day of activities facilitated through direct, intensive teacher support. It is possible that this level of support limited the number of naturally occurring opportunities for both student-to-student and teacher-to-student interactions. However, settings focused on delivering high-quality, early intervention services, such as this one, are typically arranged with similar staffing ratios. Research should focus on the use of Snack Talk in settings with decreased levels of teacher support. Specifically, exploration of peer-mediated implementations of intervention should be conducted, utilizing peers as models of implementation to extend and sustain implementations of intervention in settings with higher teacher-to-student staffing ratios.

Lastly, data presented in this study are aggregated to account for changes in staffing throughout the intervention. Further, it was classroom practice in this early intervention program to consistently switch teachers during mealtimes, making it difficult to collect data on one teacher for the duration of the study. Rather, these data demonstrate an average behavior change among numerous teachers as they rotate in and out of mealtime support. However, what these data do show is the high effects of implementation of intervention with multiple staff members, presenting a realistic data set that demonstrates generalization of implementation of this intervention among a wide range of teachers. While we acknowledge that data presented do not follow one teacher or staff member throughout the implementation of the intervention, we also think these data indicate positive outcomes of high fidelity of implementation of an easy intervention with large effects to numerous students with differing abilities and language profiles. Future research should examine the effects of this intervention with individual teachers to address this limitation.

## 5. Conclusions

Results of this study demonstrate the effectiveness of an easily implemented intervention that resulted in maximized opportunities for learning in early childhood classrooms. Snack Talk provides not only valuable social skills instruction to students of all abilities but also increased opportunities for interaction and skill building for teachers. When implemented, Snack Talk affords teachers with a simple way of embedding instruction within an existing routine previously not utilized for instruction. Snack Talk’s simple procedures enable teachers to provide embedded instruction on multiple objectives to a range of diverse learners at the same time. Further, the universal approach of Snack Talk ensured teachers were able to interact equitably with *all* students, not just students with strong verbal communication skills. The skills targeted in this intervention are life-long skills and are identified outcomes of inclusive, blended settings. This intervention has important implications for social belonging, community membership, and quality of life of children of all abilities.

## Figures and Tables

**Table 1.** Percentage of Interobserver Agreement for Each Condition.

Participants	Baseline 1 (Range)	Intervention 1 (Range)	Baseline 2 (Range)	Intervention 2 (Range)	Generalization
Corey	98% (97–100)	89% (87–92)	99% (98–100)	93% (90–97)	92%
Marie	93% (85–100)	88% (87–93)	90% (93–93)	88% (83–93)	93%
Jonathan	100%	85% (83–87)	100%	92% (92–92)	90%
Claire	97% (95–98)	91% (88–93)	98% (97–98)	100%	97%
Justin	97%	93% (92–95)	98% (98–98)	94% (92–97)	92%

*Note*. Adapted from Bateman and colleagues [7].

**Table 2.** Social Validity Questions and Results.

	Social Validity Questions	Average Rating
1. To what extent do you feel this intervention (Snack Talk) seemed effective in increasing communication (teachers or peers) during mealtimes?		5
2. To what extent do you feel this intervention (Snack Talk) seemed effective in increasing engagement during mealtimes?		4
3. To what extent do you feel that this intervention (Snack Talk) seemed effective in increasing confidence in teachers to support students with ASD during mealtimes?		4.75
4. To what extent do you feel that this intervention (Snack Talk) seemed effective in increasing interactions among children and staff during mealtimes?		4.75

*Note*. The scale was 1 = very ineffective to 5 = very effective.

## Data Availability

Data is unavailable due to privacy restrictions.
